# Metastatic breast carcinoma in the mandible presenting as a periodontal abscess: a case report

**DOI:** 10.1186/1752-1947-5-265

**Published:** 2011-07-01

**Authors:** Evmenios Poulias, Ioannis Melakopoulos, Konstantinos Tosios

**Affiliations:** 1 Department of Periodontics, University of Louisville School of Dentistry, Louisville, KY, USA; 2 Private Practice, Athens, Greece; 3 Department of Oral Pathology and Surgery, School of Dentistry, National and Kapodestrian University of Athens, Athens, Greece

## Abstract

**Introduction:**

Tumors can metastasize to the oral cavity and affect the jaws, soft tissue and salivary glands. Oral cavity metastases are considered rare and represent approximately 1% of all oral malignancies. Because of their rarity and atypical clinical and radiographic appearance, metastatic lesions are considered a diagnostic challenge. The purpose of this report is to present a rare case of a metastatic breast carcinoma mimicking a periodontal abscess in the mandible.

**Case presentation:**

A 55-year-old Caucasian woman was referred to our clinic for evaluation of bisphosphonate-induced jaw osteonecrosis. She had undergone modified radical mastectomy with axillary lymph node dissection for invasive ductal carcinoma of the left breast. Her clinical examination showed diffuse swelling and a periodontal pocket of 6 mm exhibiting suppuration in the posterior right mandible. Moreover, paresthesia of the lower right lip and chin was noted. There were no significant radiographic findings other than alveolar bone loss due to her periodontal disease. Although the lesion resembled a periodontal abscess, metastatic carcinoma of the breast was suspected on the basis of the patient's medical history. The area was biopsied, and histological analysis confirmed the final diagnosis of metastatic breast carcinoma.

**Conclusion:**

The general dentist or dental specialist should maintain a high level of suspicion while evaluating patients with a history of cancer. Paresthesias of the lower lip and the chin should be considered ominous signs of metastatic disease. This case highlights the importance of the value of a detailed medical history and thorough clinical examination for the early detection of metastatic tumors in the oral cavity.

## Introduction

Metastases in the oral cavity are rare and comprise approximately 1% of all oral malignancies [1]. They usually involve the jaws but may also be found in the soft tissues and salivary glands. The most common metastatic malignancies in women are from primary cancers in the breasts, kidneys, colorectal region, genital organs and thyroid glands, and in men they arise from the lungs, prostate, kidneys and colorectal region [2,3]. The mandible is affected more frequently than the maxilla, with a predilection for the areas distal to the canines, including the body and ramus [1,2,4]. These sites are considered vulnerable to the deposition of neoplastic cells because of the presence of hematopoietic bone marrow, branching of the local blood vessels and slowing of blood flow [4].

A wide range of clinical signs and symptoms may be seen in association with metastatic tumors of the oral cavity, with the most common being pain, swelling, altered sensation, halitosis, gum irritation, tooth loosening and mobility, exophytic masses of the soft tissues, trismus and, rarely, pathologic fractures [1,2,4]. Numbness or paresthesia of the lower lip and chin is considered an important sign of metastatic disease [5].

Metastatic tumors of the oral cavity do not exhibit a pathognomonic radiographic appearance; therefore, radiographic examination is rarely considered diagnostically important. Osteolytic radiolucent lesions with ill-defined and irregular margins may be seen, while osteoblastic lesions with a pure radiopaque or a mixed radiopaque-radiolucent appearance are typically associated with prostate cancer [2,4,6]. Early detection of jaw metastasis can be challenging. In the initial stages of the disease, the lesion may not produce a radiographic appearance. In an analysis of 390 cases of metastatic tumors of the jaw, Hirshberg *et al*. [6] found that 5.4% of them did not show any important radiographic change.

The purpose of this report is to describe a rare case of a metastatic breast carcinoma in the mandibular gingival tissue that mimicked a periodontal abscess.

## Case presentation

A 55-year-old Caucasian woman with subtle pain and tenderness in the area surrounding the right third mandibular molar was referred to our clinic by her oncologist with the provisional diagnosis of bisphosphonate-induced jaw osteonecrosis. Her medical history revealed a modified radical mastectomy with axillary lymph node dissection for invasive ductal carcinoma of the left breast. The tumor was positive for estrogen receptors and cerbB2, but negative for progesterone receptors; thus she received adjuvant hormone therapy with tamoxifen. Moreover, bisphosphonate treatment was initiated with 4 mg of intravenous ibandronic acid administered every three weeks.

**Figure 1:**
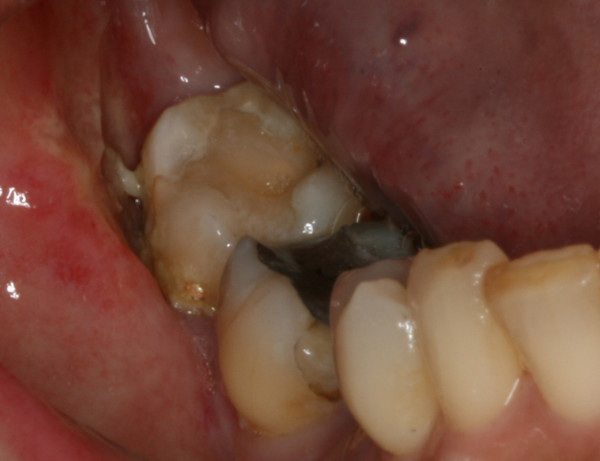
**Intra-oral view showing a diffuse swelling located over the buccal gingiva of the mandibular molar region and drainage of purulent exudate**.

An intra-oral examination revealed diffuse swelling of the buccal gingiva surrounding the second and third molar teeth that was soft and tender on palpation, with signs of inflammation (Figure 1). The involved teeth showed slight mobility, moderate plaque and calculus deposits, bled upon probing and reacted positively in repeated vitality tests. The patient's periodontal examination revealed severe generalized chronic periodontitis, with pockets in the posterior area of the right mandibular quadrant ranging from 3 mm to 7 mm in depth. A 6 mm periodontal pocket with suppuration was detected in the mesial buccal aspect of the third molar. An examination of the intra-oral area innervated by the mental nerve also revealed altered sensation, and the patient admitted paresthesia of the lower lip and chin during an extra-oral examination. Regional lymph nodes were not palpable.

**Figure 2:**
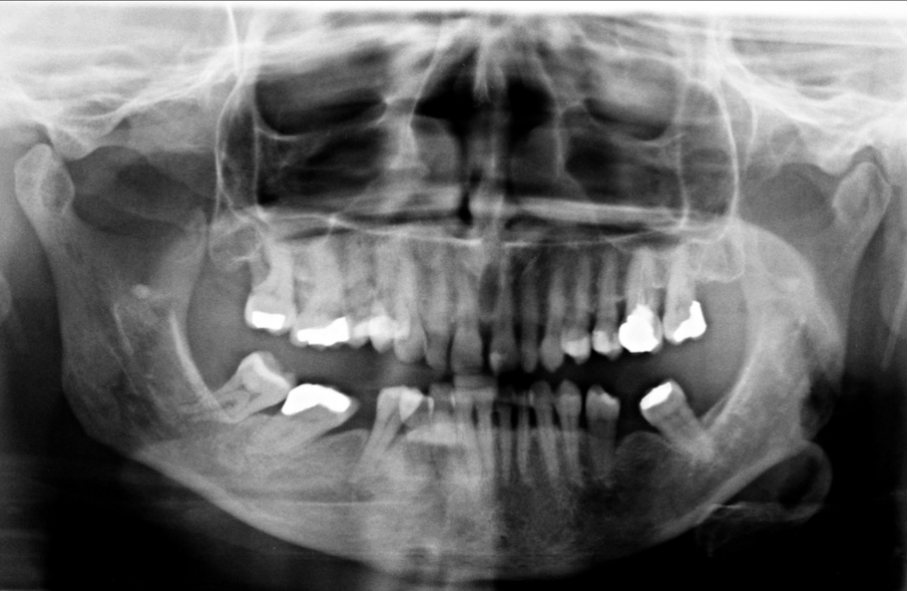
**Panoramic radiograph showing generalized bone loss throughout the dentition**.

**Figure 3:**
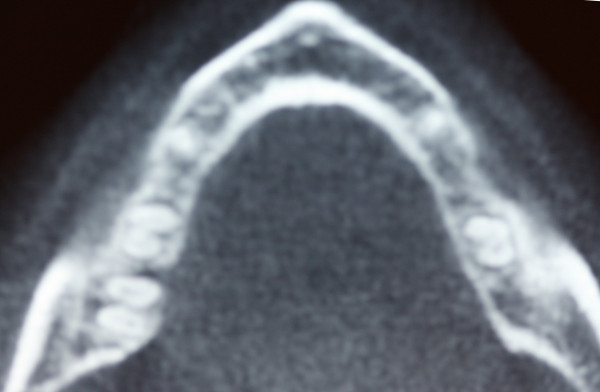
**Small radiolucent areas in close proximity with the third molar on an axial cone beam computed tomographic (CBCT) image of the mandible**.

**Figure 4:**
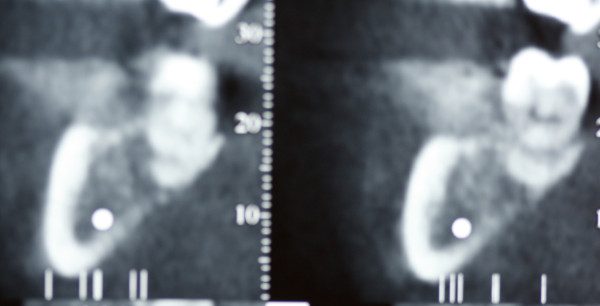
**Small radiolucent areas in close proximity to the third molar on serial cross-sectional CBCT images of the mandible**.

A panoramic radiograph showed generalized horizontal bone loss throughout the patient's dentition (Figure 2). A peri-apical radiograph of the involved area revealed alveolar bone loss attributable to the periodontal disease. Axial and serial cross-sectional 1 mm-thick cone beam computed tomography (CBCT) showed small radiolucent areas in close proximity to the third molar (Figures 3 and 4) that were not diagnostic of metastases.

On the basis of the patient's medical history and paresthesia of the lower lip and chin, metastatic disease was highly suspected. The differential diagnosis included acute or chronic periodontal abscess, acute alveolar abscess, bisphosphonate-induced jaw osteonecrosis and osteomyelitis.

**Figure 5:**
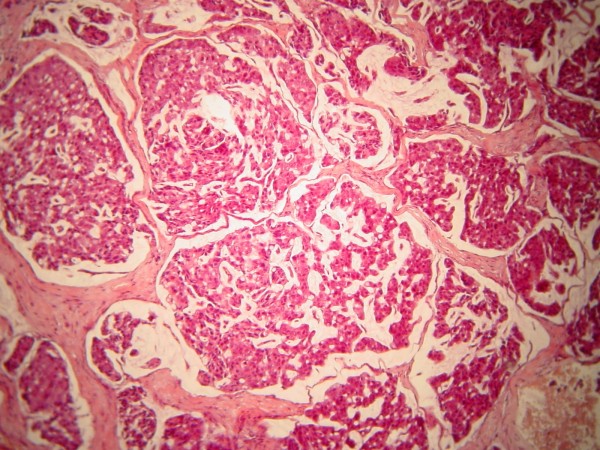
**Solid and cribriform nests of neoplastic cells in vascular, myxofibromatous stroma (hematoxylin and eosin stain; original magnification, × 200)**.

**Figure 6:**
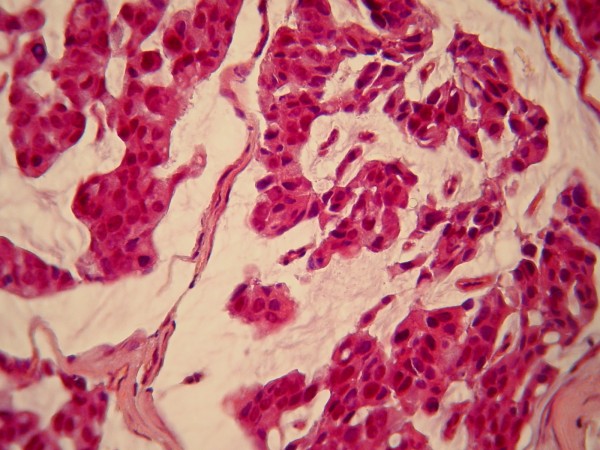
**Neoplastic cells with abundant eosinophilic cytoplasm and large, pleomorphic, darkly stained nuclei (hematoxylin and eosin stain; original magnification, × 400)**.

The swelling of the buccal gingiva was biopsied. Five-micron-thick, formalin-fixed, paraffin-embedded tissue sections stained with hematoxylin and eosin showed a fragment of parakeratinized oral mucosa infiltrated by solid and cribriform nests of neoplastic cells in a vascular and myxofibromatous stroma (Figure 5). The neoplastic cells contained abundant eosinophilic cytoplasm and large, pleomorphic, darkly stained nuclei (Figure 6). Several mitoses were observed, including atypical forms, as well as minimal lymphoplasmacytoid inflammatory infiltration of the stroma. The diagnosis was consistent with metastatic carcinoma of breast origin. Slides from the primary breast lesion were not available for comparison with the metastatic focus.

The patient was referred back to her oncologist. A full body scan did not reveal additional metastases, and a technetium-99 m-methylene diphosphonate bone scan located a region of increased radioisotope uptake ("hot spot") on the posterior right side of the mandible.

**Figure 7:**
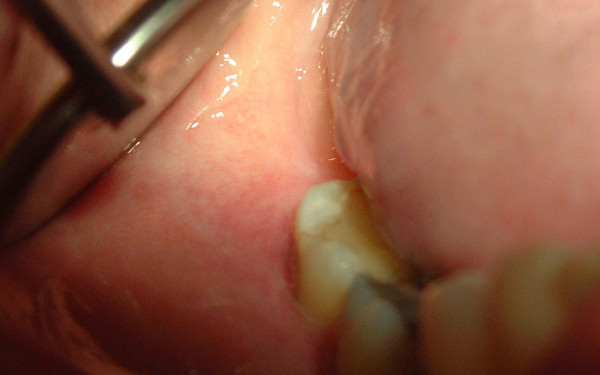
**One-year follow-up intra-oral view of the buccal gingiva of the mandibular right molar region**. No inflammatory signs were noted, and remission of the disease was achieved.

The bisphosphonate treatment was continued, and local irradiation of the right posterior mandible was administered as palliative treatment. Although extraction of the involved teeth prior to radiotherapy was feasible, it was decided to preserve them and re-evaluate their prognosis during the follow-up appointments. The patient underwent radiation therapy with a cumulative dose of 3000 cGy fractionated over two weeks, which resulted in complete relief of her symptoms and remission of the disease (Figure 7). Follow-up examinations were performed every two weeks for the first two months and bimonthly over the next two years. At the time of this writing, there is no evidence of recurrence.

## Discussion

The diagnosis of metastasis to the oral cavity is a significant challenge to the clinician because of the lack of pathognomonic signs and symptoms. To the best of our knowledge, this is the first reported case of a metastatic breast cancer mimicking a periodontal abscess. Previously described cases of metastases to the periodontal tissues were associated with extensive osteolytic destruction of the alveolar bone and root apex resorption [7-9], even in cases in which an exophytic mass was seen [10,11].

Our patient was referred to our clinic by her oncologist for the evaluation of possible osteonecrosis of the jaw caused by bisphosphonate treatment. The patient's oral cavity was carefully examined, but no signs of exposed avascular necrotic bone were found in the mandible. The existence of exposed necrotic bone over a period of eight weeks with past or recent use of bisphosphonates is an essential element for rendering the diagnosis of osteonecrosis associated with bisphosphonates, along with the absence of previous radiation therapy to the jaws [12]. Therefore, on the basis of the clinical characteristics of our patient, this type of lesion was excluded.

The local inflammation of the soft tissues that surrounded the area and the periodontal pocket exhibiting suppuration were signs of possible inflammatory reactions such as an acute or chronic periodontal abscess, an acute alveolar abscess or a combined endodontic-periodontic lesion. However, the relatively healthy condition of the patient's teeth, the existence of vital pulp after several diagnostic tests and the lack of radiographic signs eliminated the possibility of an endodontic-related lesion.

In the case presented herein, the location of the swelling, spontaneous intra-pocket suppuration and the existence of typical signs of periodontal disease were suggestive of a periodontal abscess. Periodontal abscesses are most often associated with a pre-existing periodontal pocket and present as an ovoid elevation of the gingival tissue along the lateral side of the root [13]. Depending on their location, a small or a diffuse swelling may be seen. They may also appear as erythema when they are located deep in the periodontium. The most common symptoms reported are pain and tenderness of the affected area. Drainage of purulent exudate from the periodontal pocket itself or from a fistula in the oral cavity is often noted. Other findings include increased tooth mobility, increased sensitivity to percussion, as well as, occasionally, lymphadenopathy and elevated body temperature. Radiographic examination of the periodontal abscess can vary significantly, and the findings can range from widening of the periodontal ligament to pronounced bone loss along the root of the infected tooth. Furthermore, in many cases, the radiographic examination may reveal a normal appearance of the inter-dental bone, especially when the abscess is located on the facial or lingual surfaces of the tooth [13,14]. In our patient, we decided to perform a biopsy because of the history of malignant disease and the existence of lip and chin paresthesia.

Paresthesia of the lower lip and chin is the major symptom suggestive of metastatic disease. It is described in the literature as mental nerve neuropathy or numb chin syndrome (NCS) [5,15]. The nerves associated with the NCS are the inferior alveolar nerve and its terminal branch, the mental nerve, which are branches of the third (mandibular) division of the trigeminal nerve. In addition to the chin and lip paresthesia, numbness of the teeth and mucosa may occur. Although NCS may be iatrogenic and is often caused by dental anesthesia or inferior alveolar nerve injury after improper placement of dental implants, it may also occur as the result of a benign or malignant neoplasm that disrupts the function of the nerve. Neoplasms that are most commonly associated with NCS are lymphomas and metastatic carcinomas of the mandible [15,16]. Our patient did not report paresthesia as the chief complaint, but careful intra-oral and extra-oral examinations revealed altered sensation to the lip and chin. Therefore, the existence of NCS should always alert the dentist or the physician to investigate the presence of a primary or recurrent malignant neoplasm, especially in cases that involve a significant medical history.

The management of metastatic breast carcinomas of the oral cavity is primarily palliative and may include radiotherapy, chemotherapy, hormone therapy and, rarely, surgical intervention. Pain relief and avoidance of possible infections, fractures or hemorrhage should be the major goals [17]. Local radiotherapy is almost always the treatment of choice as it relieves pain, prevents loss of function and arrests growth of the tumor [18,19]. A combination of surgical excision and radiation therapy is used in most cases of soft-tissue metastases [19].

The prognosis for patients with metastatic lesions of the oral cavity is generally poor, primarily because of the delay in the detection of the lesions. The average survival time for patients with metastatic tumors in the oral cavity is six to seven months, with approximately 70% of patients dying within one year of diagnosis [6,19,20]. Most patients with oral metastases have already developed generalized metastases by the time of diagnosis; however, in many cases, a solitary mandibular metastasis can be the initial manifestation of the primary tumor.

## Conclusion

In conclusion, this case illustrates the importance of suspecting a metastatic lesion in the jaw, despite the lack of clinical or radiographic evidence. The general dentist or dental specialist should obtain the patient's complete medical history and carefully evaluate unusual clinical and radiographic findings such as lip and chin paresthesias to differentiate metastatic lesions from clinically similar entities. As these lesions are associated with a poor prognosis, early detection is of extreme importance.

## Consent

Written informed consent was obtained from the patient for publication of this case report and any accompanying images. A copy of the written consent is available for review by the Editor-in-Chief of this journal.

## Competing interests

The authors declare that they have no competing interests.

## Authors' contributions

PE and MI analyzed and interpreted the patient data. TK performed the histological examination of the biopsy specimen and was involved in the manuscript editing and review. PE was involved in the literature review as well as manuscript preparation, editing and submission. MI was involved in the manuscript editing and review. All authors read and approved the final manuscript.

## References

[B1] Dib LL Soares AL Sandoval RL Nannmark U Breast metastasis around dental implants: a case report Clin Implant Dent Relat Res 2007 9 112 115 10.1111/j.1708-8208.2007.00033.x 17535335

[B2] D'Silva NJ Summerlin DJ Cordell KG Abdelsayed RA Tomich CE Hanks CT Fear D Meyrowitz S Metastatic tumors in the jaws: a retrospective study of 114 cases J Am Dent Assoc 2006 137 1667 1672 17138711 10.14219/jada.archive.2006.0112

[B3] Friedrich RE Abadi M Distant metastases and malignant cellular neoplasms encountered in the oral and maxillofacial region: analysis of 92 patients treated at a single institution Anticancer Res 2010 30 1843 1848 20592390

[B4] Akinbami BO Metastatic carcinoma of the jaws: a review of literature Niger J Med 2009 18 139 142 19630317

[B5] Ryba F Rice S Hutchison IL Numb chin syndrome: an ominous clinical sign Br Dent J 2010 208 283 285 10.1038/sj.bdj.2010.292 20379242

[B6] Hirshberg A Leibovich P Buchner A Metastatic tumors to the jawbones: analysis of 390 cases J Oral Pathol Med 1994 23 337 341 10.1111/j.1600-0714.1994.tb00072.x 7815371

[B7] Lu SY Chen L Mandible metastasis as the initial manifestation of breast carcinoma: report of a case Zhonghua Ya Yi Xue Hui Za Zhi 1991 10 98 103 1820829

[B8] Ogütcen-Toller M Metin M Yildiz L Metastatic breast carcinoma mimicking periodontal disease on radiographs J Clin Periodontol 2002 29 269 271 10.1034/j.1600-051x.2002.290314.x 11940148

[B9] Rawal YB Blakenship JA Mincer HH Parrish ML Anderson KM Metastatic adenocarcinoma of the breast presenting as pulpal/periodontal disease J Tenn Dent Assoc 2007 87 11 13 17539226

[B10] Rajesh KS Varma BR Bhat KM Metastasis to maxillary gingiva from carcinoma of breast: a case report Indian J Dent Res 1998 9 23 27 10530189

[B11] Alvarez-Alvarez C Iglesias-Rodríguez B Pazo-Irazu S Delgado-Sánchez-Gracián C Colonic adenocarcinoma with metastasis to the gingiva Med Oral Patol Oral Cir Bucal 2006 11 E85 E87 16388302

[B12] Ruggiero SL Dodson TB Assael LA Landesberg R Marx RE Mehrotra B American Association of Oral and Maxillofacial Surgeons position paper on bisphosphonate-related osteonecrosis of the jaws: 2009 update J Oral Maxillofac Surg 2009 67 2 12 19371809 10.1016/j.joms.2009.01.009

[B13] Herrera D Roldán S Sanz M The periodontal abscess: a review J Clin Periodontol 2000 27 377 386 10.1034/j.1600-051x.2000.027006377.x 10883866

[B14] Dahlén G Microbiology and treatment of dental abscesses and periodontal-endodontic lesions Periodontol 2000 2002 28 206 239 10.1034/j.1600-0757.2002.280109.x 12013343

[B15] Lesnick JA Zallen RD Numb chin syndrome secondary to metastatic breast disease J Colo Dent Assoc 1999 78 11 14 10686889

[B16] Harris CP Baringer JR The numb chin in metastatic cancer West J Med 1991 155 528 531 1815405 PMC1003080

[B17] Stavropoulos MF Ord RA Lobular adenocarcinoma of breast metastatic to the mandibular condyle: report of a case and review of the literature Oral Surg Oral Med Oral Pathol 1993 75 575 578 10.1016/0030-4220(93)90227-U 8488024

[B18] Khalili M Mahboobi N Shams J Metastatic breast carcinoma initially diagnosed as pulpal/periapical disease: a case report J Endod 2010 36 922 925 10.1016/j.joen.2010.01.010 20416447

[B19] van der Waal RI Buter J van der Waal I Oral metastases: report of 24 cases Br J Oral Maxillofac Surg 2003 41 3 6 10.1016/S0266-4356(02)00301-7 12576032

[B20] Hirshberg A Shnaiderman-Shapiro A Kaplan I Berger R Metastatic tumours to the oral cavity: pathogenesis and analysis of 673 cases Oral Oncol 2008 44 743 752 10.1016/j.oraloncology.2007.09.012 18061527

